# Structural analysis of cross α-helical nanotubes provides insight into the designability of filamentous peptide nanomaterials

**DOI:** 10.1038/s41467-020-20689-w

**Published:** 2021-01-18

**Authors:** Fengbin Wang, Ordy Gnewou, Charles Modlin, Leticia C. Beltran, Chunfu Xu, Zhangli Su, Puneet Juneja, Gevorg Grigoryan, Edward H. Egelman, Vincent P. Conticello

**Affiliations:** 1 Department of Biochemistry and Molecular Genetics, University of Virginia, Charlottesville, VA 22908 USA; 2 Department of Chemistry, Emory University, Atlanta, GA 30322 USA; 3 The Robert P. Apkarian Integrated Electron Microscopy Core (IEMC), Emory University, Atlanta, GA 30322 USA; 4 Department of Computer Science, Dartmouth College, Hanover, NH 03755 USA; 5 Department of Biological Sciences, Dartmouth College, Hanover, NH 03755 USA

**Keywords:** Cryoelectron microscopy, Biomaterials - proteins, Molecular self-assembly

## Abstract

The exquisite structure-function correlations observed in filamentous protein assemblies provide a paradigm for the design of synthetic peptide-based nanomaterials. However, the plasticity of quaternary structure in sequence-space and the lability of helical symmetry present significant challenges to the de novo design and structural analysis of such filaments. Here, we describe a rational approach to design self-assembling peptide nanotubes based on controlling lateral interactions between protofilaments having an unusual cross-α supramolecular architecture. Near-atomic resolution cryo-EM structural analysis of seven designed nanotubes provides insight into the designability of interfaces within these synthetic peptide assemblies and identifies a non-native structural interaction based on a pair of arginine residues. This arginine clasp motif can robustly mediate cohesive interactions between protofilaments within the cross-α nanotubes. The structure of the resultant assemblies can be controlled through the sequence and length of the peptide subunits, which generates synthetic peptide filaments of similar dimensions to flagella and pili.

## Abstract (web summary)

Peptide-based filamentous assemblies are successfully used for generation of structurally ordered materials, but their de novo design and structural characterization is challenging. Here, the authors provide a strategy for the design of self-assembling peptide nanotubes based on modifications of an arginine clasp interaction motif, and report the cryo-EM structures of seven designed nanotubes.

## Introduction

Peptide-based filamentous assemblies have been employed with great success over the past several decades for the fabrication of structurally ordered materials within the nano-scale size regime for applications in biomedicine and nanotechnology^1–3^. The molecular design of these materials has relied thus far on relatively simple sequence-structure correlations derived from structural informatics analysis of native protein folds. The mode of assembly involves self-association of structural subunits (i.e., protomers) into long non-covalent polymers that display helical symmetry. While de novo design has been employed frequently to identify suitable candidate peptides for construction of synthetic helical assemblies, the number of structures that have been characterized at near-atomic resolution is relatively limited^4–13^. As recent high-resolution structural analyses have demonstrated for both native and synthetic protein filaments, atomic models for helical assemblies based on low-resolution data are incomplete and often in error^14–17^. In the infrequent cases in which high resolution analysis has been performed on designed peptide filaments, the observed structures can differ significantly from the models that were employed as the basis for the design. These results suggest that our current knowledge of the design principles that underlie the formation of helical peptide and protein assemblies remains limited in scope, even for the simple structural motifs that have been employed as substrates in these studies. In addition, it raises the question of whether quaternary structure, i.e, interactions at the interfaces between protomers, is robust in sequence space, that is, designable^18^, and can be employed for reliable and predictable design of synthetic peptide filaments^12,19–26^.

Given the difficulties involved in correctly predicting quaternary structure, the de novo design of synthetic peptide assemblies requires validation of the predicted model through structural determination at near-atomic level resolution. This structural information is essential for understanding the inter-subunit contacts that stabilize the interfaces within filamentous peptide assemblies, especially as these interactions are critically important for functional properties such as mechanical response and bioactivity^27–29^. Structural polymorphism is frequently observed for designed helical peptide assemblies, as well as for biologically derived protein filaments that have been assembled in vitro^19–23^. Diverse populations of structurally distinct filaments can arise from the same peptide or protein sequence. Selection for monomorphic variants of defined and predictable structure represents a significant challenge to de novo design of peptide-based materials. Slight variations in preparative conditions can result in the formation of distinctly different structural variants in a manner that has yet to be understood at the level of atomic interactions^24–26^. Therefore, structural information that results from near-atomic resolution analyses of helical assemblies, yielding reliable atomic models, is essential for interrogating the limits of our knowledge of peptide design as well as for reverse engineering of structure to generate assemblies reliably and predictably.

**Fig. 1:**
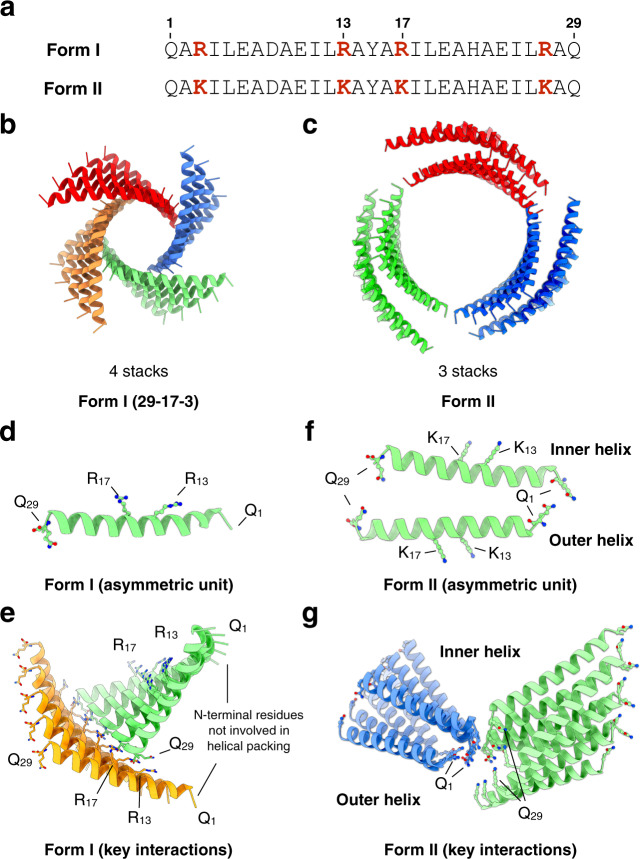
Structures and critical interactions in the Form peptide filaments. **a** The 29-residue sequences of Form I and Form II peptides. **b**, **c** Cryo-EM structure of Form I (**b**) and Form II (**c**) filaments. **d** The building block (asymmetric unit) of Form I peptide with key residues shown in sticks. **e** Essential interactions between adjacent helix stacks in Form I filaments that maintain the helical packing. **f** The building block (asymmetric unit) of the re-examined Form II peptide with key residues shown in sticks. **g** Essential interactions between adjacent helix stacks in Form II filaments that maintain the helical packing.

We recently described the structural analysis of helical filaments derived from two related synthetic coiled-coil peptides, Form I and Form II (Fig. 1 and Supplementary Fig. 1)^12^. Atomic models were generated using Rosetta modeling of the peptides into the density map derived from electron cryomicroscopy (cryo-EM) with direct electron detection^30^. Although cryo-EM has come to dominate structural biology within the past several years, and has emerged as the main technique for determining the atomic structure of macromolecular complexes^31–33^, applications of this method in chemistry and materials science have been relatively sparse^10–12,34,35^. However, the resolution revolution in cryo-EM has enabled direct structural determination of designed helical assemblies at near-atomic resolution that were previously inaccessible. While the sequences of the Form I and Form II peptides differed solely in the substitution of arginine residues in the former sequence for lysine residues in the latter sequence (Fig. 1a), the corresponding atomic models for the filaments displayed significant differences in helical symmetry and the nature of the cohesive interactions between protomers. These differences were not anticipated in that the corresponding sequence substitutions occurred at positions that were not expected to participate directly in interfacial interactions (Supplementary Fig. 1)^12,30^. Moreover, site-directed mutagenesis of one or two residues within the respective sequences of Form I and Form II resulted in interconversion between the two alternative helical structures (vide infra). While the structural transition could be rationalized on the basis of the differences between the atomic models of the respective filaments, neither structure was predicted a priori, nor could the structures be considered as robust in sequence space, due to the significant impact of limited mutagenesis. Indeed, the dramatic change in quaternary structure due to the semi-conservative mutagenesis of one or two amino acids is suggestive of nearly chaotic behavior.

Here we report seven high-resolution cryo-EM structures of filamentous peptide nanotubes based on a cross-α supramolecular architecture that provide insight into the designability of helical peptide filaments. In addition, emergent structural behavior is observed among the corresponding helical filaments. A structural interaction is identified based on an appropriately placed pair of arginine residues (RxxxR) within the corresponding peptide sequences. This local interaction motif, designated as an arginine clasp, is robust within the context of these cross-α assemblies and can guide the coalescence of helical protofilaments into structurally defined cross-α nanotubes. Despite this observation, the arginine clasp motif does not appear to have been evolutionarily sampled as a mechanism to mediate similar helix-helix interactions in naturally occurring protein structures and, therefore, cannot be considered as natively designable.

## Results

### Structural differences between the Form I and Form II filaments

The respective assemblies of Form I and Form II epitomized an unusual packing motif, recently designated as a cross-α structure^36^ (alternatively, an α-sheet structure^37^). In contrast to most structurally characterized filaments based on synthetic α-helical coiled-coils^38–46^, the chain axes of the helical protomers are oriented in a plane nearly perpendicular to the long axis of the assembly. The initial reconstruction of Form II resulted in an atomic model of relatively low (circa 7-Å) resolution, in contrast to the higher (3.6-Å) resolution of the corresponding model for Form I (PDB ID: 3J89, [https://www.rcsb.org/structure/3j89]). In order to better compare the two models, cryo-EM data were collected on a fresh preparation of Form II filaments (Fig. 1c). Helical reconstruction afforded an atomic model at 4.2-Å resolution with a different helical symmetry (i.e., a rise of 1.93 Å and a rotation of 124.4°) than that proposed for the original model^12^. The higher resolution model of Form II is consistent with a parallel orientation of the inner-outer helix pair, which was suggested but could not be unambiguously determined in the previous analysis^12^.

A structural comparison of the two assemblies indicated that helical protomers were arranged in four (Form I) or three (Form II) cross-helical stacks (i.e., protofilaments) that self-associated laterally to form single-walled and double-walled nanotubes, respectively (Fig. 1). The axial interactions along the respective stacking interfaces were conserved between the different filaments. These interfaces were stabilized through a series of heterotypic knobs-into-holes (KIH) interactions between hydrophobic residues at the a/d and c/f faces of axially adjacent helices within a protofilament (Supplementary Fig. 1)^12,37^. The main distinguishing feature between the Form I and Form II assemblies was the mode of lateral association between the respective cross α-helical stacks. The Form I structure displayed a unique interaction involving a pair of arginine residues, R13 and R17, that formed a network of hydrogen bonds and electrostatic interactions with the C-terminal residues of helices in an adjacent protofilament (Fig. 1e). Mutagenesis of either of these residues to lysine resulted in conversion of the assembly to a Form II-like structure^12^. Conversely, a K13R, K17R double mutant of the Form II sequence resulted in conversion of the resultant assembly to a Form I-like structure, which suggested that the presence of both arginine residues was necessary for formation of the Form I structure. High-resolution cryo-EM structural analysis led to identification of the critical interactions that were responsible for the structural differences between the Form I and Form II filaments, as well as the ability to directly interrogate the importance of these interactions through site-directed mutagenesis.

These results suggested that the RxxxR structural unit, in which the two arginine residues, R13 and R17, are arranged as i,i + 4 within the Form I sequence, defined a side-to-end helical interaction motif within the assembly^47^. While interactions involving individual RxxxR motifs may be relatively weak, many such interactions occurred at the lateral interfaces between protofilaments along the contour length of the Form I structure. Consequently, the influence of these localized interactions on the stability of the helical assembly was magnified significantly. Numerous instances of the stabilizing effect of structural interactions between appropriately substituted i,i + 4 residues have been described within the sequences of helical peptides^48–57^. In addition, specific interaction motifs such as the left-handed leucine zipper, i.e., LxxxLxx^58–60^, and the right-handed glycine zipper^61–63^, i.e., GxxxG, have been observed to promote and stabilize lateral (side-to-side) interactions in coiled-coils and transmembrane helical bundles, respectively^64^. The crossing angle, Ω, between laterally interacting pairs of helices in the Form I structure was circa 94°. In contrast, the leucine and glycine zipper motifs define side-to-side association between helices and display more acute crossing angles (Ω = -20° and +40°, respectively)^58,63,65^. PISA^66^ analysis indicated that the lateral interface between interacting helices in the Form I filament buried approximately 275 Å^2^/peptide. For comparison, the coiled-coil interaction along the axial direction of the Form I protofilaments defined an interface of approximately 700 Å^2^ of surface area/peptide with a helix crossing angle of circa –11.5°. We designated this RxxxR interaction motif as an arginine clasp, since it mediated a distinctive local interaction between appropriately oriented protomers on structurally adjacent protofilaments.

### Dependence of filament structure on peptide length

**Fig. 2:**
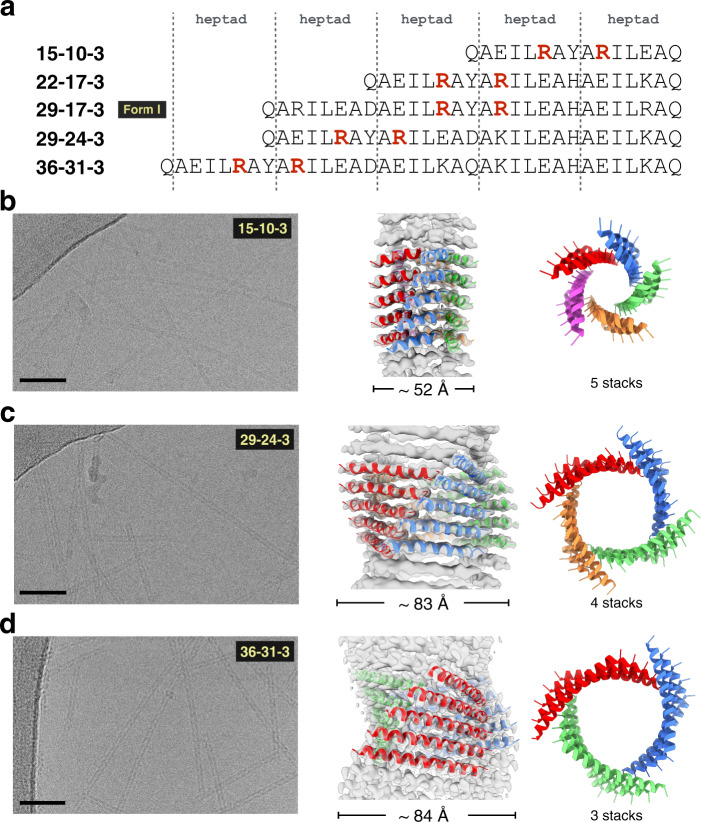
Cryo-EM of arginine clasp peptide filaments based on Form I. **a** Sequences of the Form I peptide and four designed peptides derived from Form I. **b**–**d** Cryo-EM of peptides 15-10-3, 29-24-3, and 36-31-3. Representative raw micrographs are shown on the left (scale bar 500 Å) out of a total number of 84, 279, and 314 images recorded, respectively. The cryo-EM reconstructions are shown in the middle, with models fitted into the maps. The top views of the atomic models are shown on the right.

In order to test the utility of the arginine clasp in mediating interactions within a defined structural context, we designed a series of peptides derived from the Form I sequence (Fig. 2, Supplementary Figs. 2 and 3). In the latter structure, the N-terminal heptad sequence, upstream of the RxxxR motif, was unstructured and did not contribute to the helix-helix interactions. We reasoned that the major contribution to the structural integrity of the helical filament was the region in Form I sequence space from the RxxxR motif to the C-terminus of the peptide. The corresponding peptide segment contained the critical structural elements responsible for the lateral and axial interactions. Therefore, the designed series of Form I-like peptides encompassed minimal peptide sequences in which the structurally determinative distance between the RxxxR motif and the C-terminus was systematically varied over the range from 2 to 5 heptad repeats (Fig. 2). In each case, the most N-terminal heptad contained the RxxxR motif and, in order to prevent competition, lysine residues occupied all of the other basic amino acid sites at solvent-contacting b- and e-positions within the respective heptad sequences. Lysines contribute to stabilizing electrostatic interactions with glutamic acid residues that occur between peptides within a cross-α stack, but minimally to the cohesive interactions between stacks^12^. The nomenclature of the peptides was changed to one that reflected the structural elements that were considered to be critical to the design. All peptides were designated as (x-y-z), in which x refers to peptide length, y refers to the number of residues from the first arginine to the C-terminus, and z to the number of residues between the two arginines (Fig. 2). The original Form I sequence corresponded to peptide 29-17-3 under this system of nomenclature.

**Fig. 3:**
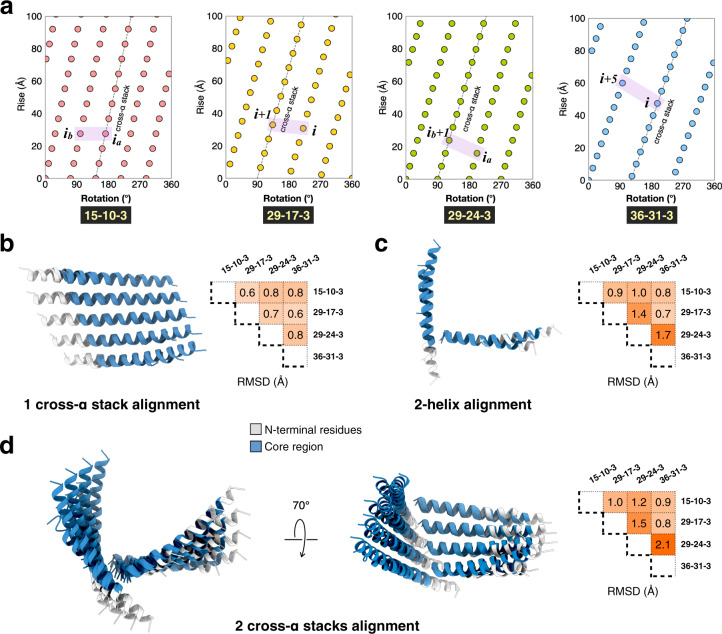
Structural conservation of the Form I-based arginine clasp filaments. **a** Helical nets for the peptide 15-10-3, 29-17-3 (Form I), 29-24-3, and 36-31-3 filaments. The helical nets show the unrolled surface lattice viewed from the outside of the filament. One of the right-handed n-start helices–associated with a cross-α protofilament within the respective assembly–is indicated with a straight line in the corresponding helical net diagram. Adjacent helices shown in **c** with the conserved interactions are highlighted in a purple box. **b** Structural alignment of a single cross-α stack containing five helices (left) and their Cα RMSD (right). **c** Structural alignment of two adjacent helices (left) and their Cα RMSD (right). **d** Structural alignment of two cross-α stacks (left) and their Cα RMSD (right).

The designed peptide sequences were based on the assumption that the helical symmetry of the respective filaments and, consequently, the axial and lateral interfaces of the Form I filament, would be conserved in the resultant assemblies. In this scenario, the RxxxR motifs within a given protofilament would interact selectively with the C-terminal residues within adjacent helices oriented (i + 1) along a left-handed 1-start helix oriented similarly to that of the Form I filament (Figs. 1b and 3a). If a given peptide self-associated into a Form I-like structure, the diameter of the resultant nanotubes would depend on the length of the peptide segment from the first arginine to the C-terminus (i.e., the y-value). The Form I assembly displayed a nearly square cross-section, in which the nanotube diameter was determined by the seventeen amino acid length of the interacting peptide segments within a laterally adjacent pair of protofilaments within the assembly (Fig. 1). If the Form I-like structure were retained, then the width of the assembly should be predictable from the position of the arginine clasp motif and the length of the peptide, as these parameters determine the cross-sectional dimensions of the corresponding assemblies. If the lengths of the interacting peptide segments were shorter than for Form I, a narrower nanotube would result and, if longer, the corresponding nanotube should display a wider diameter.

All four peptides in the (x-y-3) series adopted an α-helical conformation and formed thin filaments in aqueous buffers (Supplementary Figs. 4 and 5). Three filaments, derived from peptides 15-10-3, 29-24-3, and 36-31-3, were selected for further analysis using cryo-EM imaging with direct electron detection. The filament derived from 22-17-3 was not analyzed further, as it was expected that the structure would be similar to the previously described Form I/29-17-3, since it corresponded to a truncation of the unstructured N-terminal heptad. The iterative helical real space reconstruction (IHRSR) algorithm^67–70^ was employed to generate 3D reconstructions at final resolutions (Supplementary Table 1) of 4.2-Å, 4.1-Å, and 4.0-Å for the 15-10-3, the 29-24-3, and the 36-31-3 filaments, respectively (Fig. 2b). Surprisingly, the number of protofilaments (i.e., cross-α helical stacks) within the nanotubes was found to have an inverse relationship to peptide length. Five protofilaments interacted to form the 15-10-3 filament, four interacted in the 29-24-3 filament, and three interacted in the 36-31-3 filament. The Form I/29-17-3 structure fits the observed trend, in that the 17 amino acid length of the structurally determinative segment lay between the 10 amino acid and 24 amino acid segment length of 15-10-3 and 29-24-3, respectively.

High-resolution structural data enabled a detailed comparison between the arrangement of peptides within the respective atomic models (Fig. 3). In each case, the α-helical protomers within a protofilament stacked along a right-handed n-start helix (Fig. 3a), in which the value of n corresponded to the number of protofilaments within a nanotube. For example, each 5-start helix of 15-10-3 passed through every fifth protomer and therefore corresponded to the axial stacking direction in a protofilament (Figs. 2b and 3a). Similarly, the 4-start helices of 29-17-3 and 29-24-3 and the 3-start helices of 36-31-3, passing through every fourth subunit and every third subunit, respectively, were associated with the axial stacking direction of the protofilaments within the corresponding assemblies. The arrangement of protofilaments within the respective assemblies can be clearly discerned in the corresponding helical net diagrams (Fig. 3a). The right-handed curvature of the protofilament derived from a two-residue progressive displacement between successive helical protomers along the rise of the n-start helices. This displacement was a consequence of a heterotypic coiled-coil interaction along the stacking interface between the two offset hydrophobic faces (a/d and c/f) of the protomers, as predicted by Walshaw and Woolfson for type III coiled-coil structures (Supplementary Fig. 1)^37^. Structural overlays of helices within a protofilament (Fig. 3b) showed reasonably good alignment (root mean square standard deviation (RMSD) < 1 Å), which presumably resulted from the constraints of KIH packing at the coiled-coil interfaces despite differences that were observed at the lateral packing interfaces (Fig. 3c, d).

**Fig. 4:**
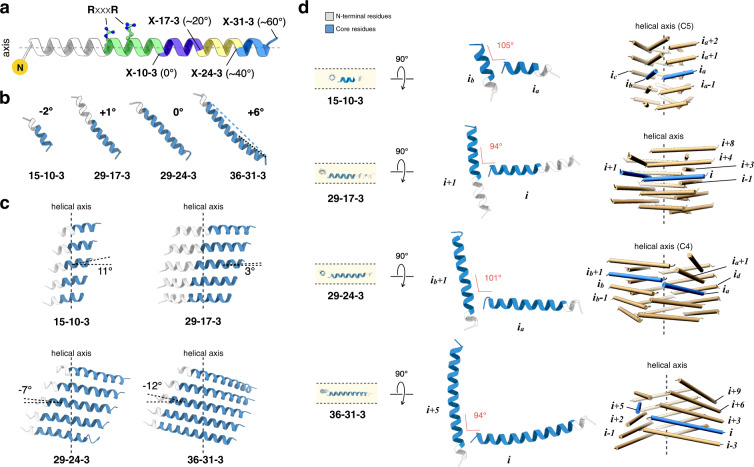
Three degrees of freedoms to maintain the conserved arginine clasp contacts. **a** The building blocks of four types of peptide filaments. The N-terminal residues not involved in filament packing are colored white. The RxxxR motif is highlighted. The core region of X-10-3 peptide is colored in green. The core region of X-17-3, X-24-3, and X-31-3 have one (magenta), two (yellow) or three (blue) additional heptads, respectively. **b** The helix tilt angle of the core region in four types of peptide filaments. **c** The cross-α stack within the respective protofilaments, indicating tilt angle from the plane normal to the helical axis. **d** Two adjacent helices that make the key contacts in these peptide filaments. The adjacent helices are oriented to be in the same plane (left), and the angle between the two helices are indicated (middle). The relative positions of these two adjacent helices (blue) in the filaments are shown (right).

Structural comparison of the filaments suggested that three mechanisms could be distinguished through which the packing of peptides adjusted itself in order to accommodate the increase in peptide length (Fig. 4). First, the tilt angle of the core peptide with respect to the plane normal to the filament axis became progressively more negative with increasing peptide length (Fig. 4c). Despite the differences in cross-sectional geometry for the respective filaments, the helix crossing angles were remarkably similar and lay within the range from 95° to 105° (Fig. 4d). We postulate that the arginine clasp interaction required that the crossing angle was within this range in order to effectively interact with the C-terminus of an adjacent helix. Adjustment of the tilt angle of helical protomers provided a mechanism to satisfy this geometric constraint. Second, for the longer peptide sequences, the lateral interaction between protofilaments could not be maintained between nearest neighbor helices (Figs. 3a and 4d). For the 29-24-3 peptide, the interaction occurred between the C-terminus of peptide (i_a_) and the RxxxR motif of peptide (i_b_ + 1), in contrast to the C5-symmetric structure of 15-10-3 that involved helices i_a_ and i_b_. Similarly, the filament structure of 36-31-3 revealed that the C-terminus of peptide (i) interacted with the axially offset RxxxR motif of peptide (i + 5) within the 1-start (C1) symmetry of the assembly. The length and flexibility of the arginine side-chains enabled the conservation of the RxxxR lateral interaction between protofilaments despite differences in peptide length. Finally, while the local helix crossing angle associated with the arginine clasp interaction was maintained at 94°, the distal segments of the 36-31-3 helices bend at a more acute crossing angle of 80° in order to maintain the overall helical symmetry of the filament. These observations suggested that peptide length determined the helical symmetry of the respective cross-α nanotubes through selection of number and relative orientation of protofilaments that could maintain the structurally critical cohesive interactions at the axial and lateral interfaces (Fig. 2).

### Effect of arginine position

The two arginine residues in the RxxxR clasp motif were positioned on the same side of the helix in an i,i + 4 orientation. This placement enabled lateral association between protofilaments in the structures of the cross-α nanotubes described above. However, a structural arrangement in which two arginines are placed in an i,i + 3 orientation, that is, as an RxxR motif, could potentially promote a similar interaction. The two arginines in the RxxR motif remain on the same face of the helical protomer, but would be arranged in a different orientation (angular displacement = -60° and axial displacement = 4.5 Å) in comparison to the RxxxR motif (angular displacement = +40° and axial displacement = 6.0 Å). Previous research demonstrated that appropriately selected residues in an i,i + 3 orientation could mediate stabilizing intra- and inter-helix interactions^48,57^.

**Fig. 5:**
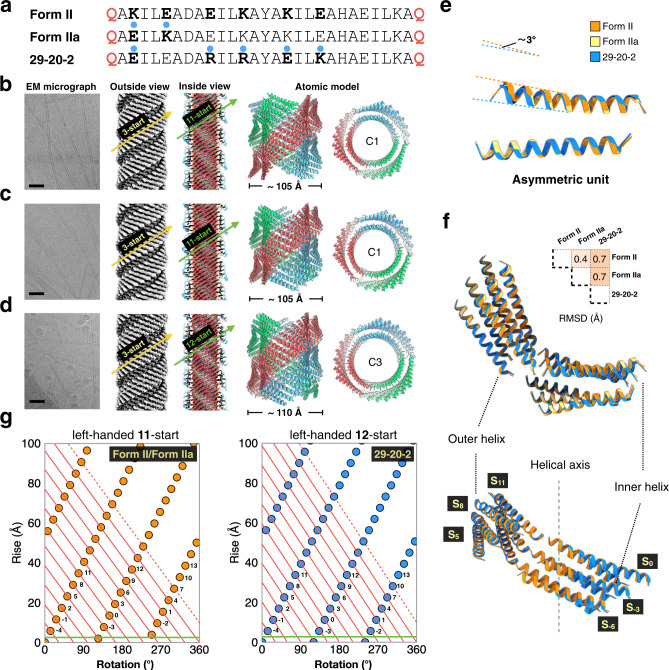
Structural comparison of the Form II-like peptides. **a** Sequences of Form II, Form IIa and 29-20-2 peptides. Residues changed from original the Form II sequences are highlighted with blue dots. **b**–**d** Cryo-EM of peptides Form II, Form IIa, and 29-20-2. Representative raw micrographs are shown on the left (scale bar 500 Å) out of a total number of 171, 233, and 347 images recorded, respectively. The cryo-EM reconstructions are shown in the middle, one view from outside highlighting right-handed 3-start helices, and another view from the inside of the nanotube lumen highlighting the left handed 11-start or 12-start helices. The side and top views of the atomic models are shown on the right. **e** Structural alignment of the asymmetric unit (helix-dimer) of the three peptide filaments. **f** Structural alignment of six asymmetric units of the three peptide filaments. The alignment RMSD is shown. **g** Helical nets for the Form II and 29-20-2 filaments. The helical nets show the unrolled surface lattice viewed from the outside of the filament. The right-handed 3-start helices can be clearly discerned for each assembly, although not explicitly highlighted. The positions of the left-handed 11- and 12-start helices are identified and are defined by the red lines crossing the green horizontal line. Subunits labeled n and n + 11 are connected with red lines.

The Form I sequence was modified to test the effect of arginine placement on the structure of the corresponding peptide assemblies (Fig. 5). Arginines were placed in an i,i + 3 (RxxR) arrangement within a heptad repeat located proximal to the N-terminus. The sequence at the axial interface was conserved in order to maintain the cross-α stacking interactions within a protofilament. The resultant peptide was designated as 29-20-2 using the same (x-y-z) nomenclature described above, with the value of **z** equal to 2 for the RxxR motif (Supplementary Figs. 6 and 7). The 29-20-2 peptide adopted an α-helical conformation in solution and. formed high aspect-ratio tubular filaments (Supplementary Figs. 8 and 9). However, the observed widths of the filaments were significantly larger, circa 12 nm, than the corresponding values for peptides in the (x-y-3) series. A 3.8-Å resolution helical reconstruction from the corresponding cryo-EM images afforded an atomic model for the 29-20-2 filament (Supplementary Table 2). Unexpectedly, the filament formed a double-walled cross-α nanotube that was similar in structure to that of the Form II filament and a structurally related mutant (Fig. 5). Remarkably, the arginine residues in RxxR motif were not involved in the lateral interactions between cross-α helical protofilaments. Instead, cohesive interactions were mediated through hydrogen-bonding between the N- and C-terminal glutamine residues from helices on laterally adjacent protofilaments, as in the Form II-related structures (Fig. 1g).

Each nanotube was derived from coalesence of three protofilaments derived from the dimeric protomer. However, these were arranged in different symmetries; C3 for 29-20-2 and C1 for Form II. In either case, the hydrophobic stacking interactions were oriented along the direction of the three 3-start helices of the respective nanotubes and coincided with the cross-α protofilaments (Fig. 5g). Among all three structures, the helices within the dimeric asymmetric unit could be superimposed with only minimal differences (Fig. 5e). Structural overlays of laterally interacting protofilaments indicated a conservation of the interfaces between the three filament structures (Fig. 5f). The main difference between the structures was manifested in terms of the lateral interactions between the termini of the protofilaments. These interactions occurred along the direction of the left-handed 12-start and 11-start helices for 29-20-2 and Form II, respectively, as illustrated in the the helical net diagrams of the corresponding assemblies (Fig. 5g). This lability of helical symmetry is common among filamentous peptide assemblies and has been observed even for structures in which the interfacial interactions between protomers are largely conserved. These differences can arise solely as a result of minor changes in interfacial packing orientation between protomers in the filament^10,17,23,71–73^.

The structural analysis of the 29-20-2 filament provided evidence that the formation of the arginine clasp interaction required not only the presence of the two arginines, but also the correct orientation, RxxxR, of the corresponding residues within the sequences of the respective helical protomers. The relatively minor substitution of an RxxR motif for an RxxxR motifs drove the lateral association down an alternative pathway that resulted in formation of a double-walled nanotube in which lateral interactions occurred between terminal glutamine residues. The latter interaction must be relatively weak as it can only sustain lateral association for a peptide having this single length. In contrast, the RxxxR motif could mediate lateral interactions over a wider range of peptide lengths from 2 to 5 heptad repeats. However, the axial interactions along the contour length of a protofilament for the Form II-like assemblies appeared quite robust and comparable in energy and buried surface area to that of Form I protofilaments.

### Native designability of the helical interfaces

**Fig. 6:**
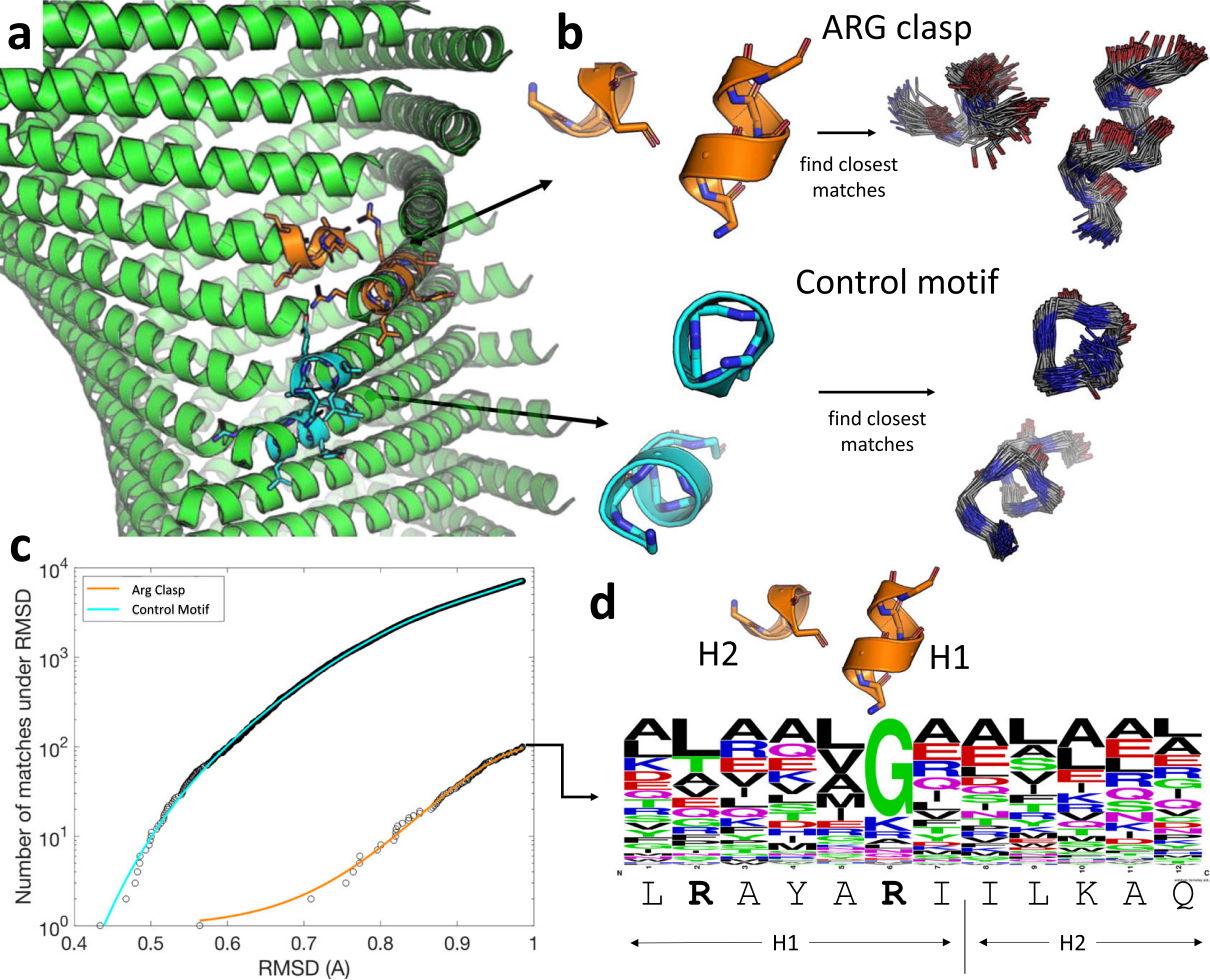
Structural analysis of arginine clasp designability. **a** The 36-31-3 assembly showing the definition of the motif used to interrogate for the presence of the arginine clasp in native structures (orange), as well as a control motif of the same size and complexity (cyan). **b** Structural alignments of resulting 100 closest matches for both motifs. The control motif, corresponding to the helix stacking interaction within a protofilament, clearly corresponds to a well-populated structural attractor, while the arginine clasp motif brings in much more diverse hits. **c** RMSD divergence plots, showing the number of matches below a given RMSD cutoff for both motifs. Matches to the Arg clasp motif are rare as the RMSD required to get up to a certain number of matches grows much more rapidly than for the control. **d** Sequence logo of the closest non-redundant sequence matches to the Arg clasp fragment, showing the query sequence underneath. The native statistics do not bear out the Arg clasp sequence motif, and the one conserved position corresponds to a Gly rather than an Arg. This preference is due to close helix-helix interactions (see Supplementary Fig. 10) and represents a different structural circumstance than the close-to-right angle docking of one helix into another, as seen in our assemblies.

The conserved structural context of the RxxxR motif in the Form I-like assemblies raised the question of its native designability, i.e., whether it occurred as a structural feature within natural proteins in which the helices were oriented similarly to those participating in the lateral interactions of Form I-like filaments. To address this question, we defined a local lateral interface motif from each of the filament structures solved here, and identified sub-structures in the PDB that displayed similar backbone geometries. The results of this analysis are reported for the 36-31-3 filament (Fig. 6), although similar results were obtained for the other Form I-like assemblies. The motif comprised a seven-residue helical fragment containing the RxxxR motif and a five-residue fragment from the C-terminus of a laterally interacting helix (Fig. 6a). Despite this compact size, the closest matching geometries from the PDB for this lateral interaction exhibited root mean squared deviations (RMSDs) over backbone atoms from the query between 0.6–0.8 Å. Furthermore, even these were rare geometries, as the RMSD of subsequent closest matches rose quickly (Fig. 6c). To demonstrate the rarity of this geometry, we compared these statistics with those of a control motif of equal complexity, corresponding to a seven-residue helix fragment, as described above, but a different five-residue helix fragment from an axially adjacent partner within the same protofilament (Fig. 6a). In all cases, this reference motif gave much more robust statistics, with many low-RMSD matches and sequence preferences consistent with the amino-acid choices in the assembly. The latter results suggested that the stacking interactions within a protofilament were locally consistent with typical helix-helix interactions, which implies that the axial helix stacking interactions are well represented in naturally occurring proteins and, therefore, are natively designable. On the other hand, the sequence statistics of the closest hits to the lateral interaction motif did not reveal a strong RxxxR signal. The only strongly conserved position corresponded to the second Arg, but the sequence logo analysis indicated that its overwhelming preference was for Gly (Fig. 6d). Upon closer inspection, we found the nature of this preference to be entirely unrelated to the arginine clasp. Rather, some of the closest matches arose in the context of an internal helix-helix interaction between two closely-approaching helices, necessitating a Gly at the position above (Supplementary Fig. 10). The results suggested that the arginine clasp motif, which involves a helix terminus interacting with the side of another helix at a nearly 100° angle, was not common within the structures of naturally occurring proteins and, consequently, not natively designable.

**Fig. 7:**
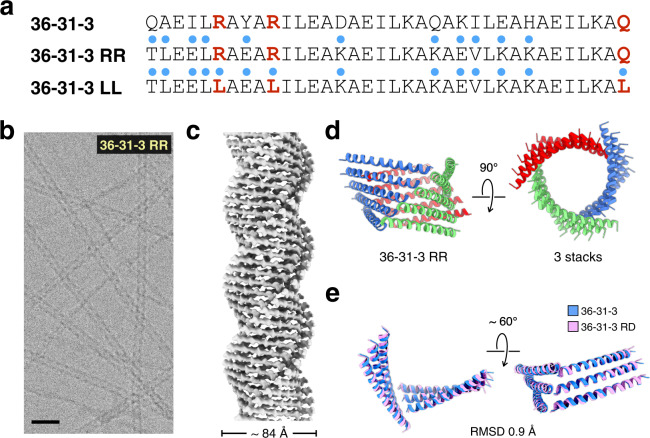
Cryo-EM of the computationally re-designed (36-31-3) peptide nanotube. **a** Sequences of the computationally redesigned peptides 36-31-3_RR and 36-31-3_LL in comparison to the parent sequence. Residues changed from original the 36-31-3 sequences are highlighted with blue dots on top. **b** Representative cryo-EM image of 36-31-3_RR peptide filaments out of a total number of 166 images recorded. Scale bar is 20 nm. **c** Cryo-EM reconstruction of the 36-31-3_RR filaments. **d** Side view and top view of the atomic model. **e** Alignment of two adjacent cross-α stacks between 36-31-3 (blue) and 36-31-3_RR (magenta).

This outcome led us to speculate whether structural motifs other than RxxxR could mediate the lateral interaction between helical stacks and promote nanotube formation. The 36-31-3 filament was employed as the starting point for a computational optimization using the design engine dTERMen^74,75^. A solubility constraint was applied in which the occupancy of the solvent-contacting b/e/g residues within the heptad repeats was constrained to polar residues. Computational optimization returned a sequence in which the RxxxR motif was replaced with an LxxxL motif and the C-terminal glutamine was replaced with a leucine residue (Fig. 7a). In the corresponding model, the network of hydrogen-bonding interactions at the lateral interface was replaced with hydrophobic interactions. In a control experiment, a second computational optimization was performed in which positions 6, 7, 10, and 36 were constrained to those in the original peptide sequence. The arginine residues at positions 6 and 10 defined the RxxxR motif, while residues at positions 7 and 36 (Ala and Gln, respectively) appeared to be important for organizing this motif. A comparison between the two sequences, 36-31-3_LL and 36-31-3_RR, could provide additional insight into importance of structural preservation of the arginine clasp interaction. Even in the absence of explicit sequence constraints, the resultant computational models retained most of the original amino acid residues at the a/d and c/f positions that mediated the heterotypic hydrophobic interaction at the axial stacking interfaces of the protofilament (Fig. 7a). Coupled with the structural similarity of axial stacking interactions between the Form I-like and Form II-like assemblies, these results provided further support that the helix-helix interactions along the axial interface were natively designable (Fig. 6c).

The computationally designed peptides, 36-31-3_LL and 36-31-3_RR, displayed an α-helical conformation and formed high aspect-ratio filaments of similar width to those of 36-31-3 (Supplementary Figs. 2–5). However, filaments derived from the 36-31-3_LL peptide unraveled into individual protofilaments over time (Supplementary Fig. 5e). In contrast, 36-31-3_RR filaments persisted over weeks in solution (Fig. 7b and Supplementary Fig. 5f). The differences in stability between the two computationally designed filaments suggested that the cohesive interactions were much weaker in the absence of the arginine clasp interaction. The most probable explanation resided in the replacement of the hydrogen-bonding network of the arginine clasp interaction within 36-31-3_RR with the network of hydrophobic interactions within 36-31-3_LL. In order to confirm that the arginine clasp was conserved, filaments of 36-31-3_RR were analyzed by cryo-EM. A 4.5-Å resolution reconstruction of the 36-31-3_RR filaments displayed a nearly identical structure to that of the original 36-31-3 filaments, with very slight differences in the rise (2.49 versus 2.51 Å/subunit) and the rotation (124.5 versus 124.0 degrees/subunit) for the respective 1-start helices of the 3D models. Accordingly, repeating the statistical analysis of this assembly’s RxxxR motif produced very similar results as for the original 36-31-3 filament, indicating the native rarity of the geometry.

## Discussion

Tayeb-Fligelman et al., reported the initial example of a cross-α fibril structure from crystallographic analysis of PSMα3, a short cytolytic peptide secreted from virulent strains of *S. aureus*^36^. The helices were observed to pack in a stacked bilayer array, in which individual peptides were oriented perpendicular to the long axis of the crystallographically defined filament. In contrast to the cross-α nanotubes described here, translational symmetry restricted coiling of the cross-α filaments. Moreover, the composition of the hydrophobic interface was rich in phenylalanine residues and did not display the KIH packing characteristically associated with coiled-coils. Subsequently, Zhang et al.^7^, reported a series of peptides based on designed coiled-coil sequences that assembled into filaments. Crystallographic analysis demonstrated that the peptides formed cross-α super-helical arrays through stacking of parallel dimers in an alternating antiparallel orientation. The filaments displayed limited interfaces over which KIH packing was observed between protomers, in contrast to the cross-α protofilaments in the nanotubes that we have described. Supercoiling of filaments was only observed under conditions in which the cross-α orientation was not maintained in the extended structure. In addition, our cross-α nanotubes differed from classical coiled-coils, in that the helical packing in a protofilament displayed an open or extended mode of association that is reminiscent of solenoidal tandem repeat proteins^10,11,76,77^. Most coiled-coils form closed assemblies of defined oligomerization state, which are often, but not exclusively, based on cyclic symmetry in which the helical protomers are aligned nearly parallel to the super-helical axis^78,79^. Surprisingly, the closest structural analogs of these cross-α nanotubes were observed among helical supramolecular assemblies derived from oligourea foldamers having similar polar patterning based on pentad repeat sequences^80^. Crystallographic characterization of the latter assemblies revealed that the oligourea nanotubes were composed of protofilaments corresponding to stacked helical subunits that propagated along right-handed 2-start or 6-start helices.

Cross-β assemblies^81^ represent a natural point of comparison to the cross-α nanotubes. The respective protofilaments differ in that cross-β architecture is primarily stabilized through main-chain hydrogen-bonding interactions along the contour length of the assembly. In contrast, the cohesive interactions between protomers within the cross-α protofilaments depended exclusively on side chain interactions. Surprisingly, few near-atomic resolution structures of synthetic cross-β nanotubes derived from designed β-strand sequences have been reported, presumably due to the propensity for structural polymorphism and concomitant difficulty in isolation of monomorphic variants^24–26^. However, the formation of cross-β nanotubes of uniform diameter has been reported for conformationally constrained cyclic peptide sequences^8,82^. More recently, cryo-EM helical reconstruction of Orb2 filaments from *D. melanogaster* brain revealed a minimal diameter nanotube composed of three identical cross-β protofilaments in a C3-symmetric arrangement^83^. Individual cross-β protofilaments were based on β-hairpin protomers interacting through a combination of main-chain and side-chain hydrogen-bonding interactions. Similar to the cross-α nanotubes, side-chain hydrogen bond formation provided the cohesive interactions between protofilaments that stabilized the cross-β assembly. These results suggested that directional non-covalent interactions, e.g., the formation of complementary hydrogen-bonded networks or metal ion coordination^84,85^, could provide a mechanism for rational design of defined higher-order structure in synthetic peptide and protein assemblies.

The de novo design of peptide and protein assemblies requires not only the control of interfacial interactions, but also the folding energetics of the individual subunits^10,18^. In this study, the protomer sequences were based on the highly designable coiled-coil motif, which has been demonstrated to display robust folding energetics and structural stability in sequence space. The axial stacking interactions within a protofilament were highly conserved between assemblies differing in both the number of protofilaments and nature of the lateral interactions between protofilaments. The structure of the axial interfaces within a protofilament were consistent with helix-helix interactions in canonical coiled-coils, even though the open-ended supramolecular architecture of the helical assemblies was quite distinct from the closed cyclic oligomers that are typically associated with such helical bundles. In contrast, the lateral interactions between protofilaments have not been commonly observed within the structures of native proteins having similar helix-helix orientations. Notably, we corroborated that the lateral interface between Form I-like protofilaments was based on the unusual interaction of an RxxxR motif with the C-terminal residues of a helix in a structurally adjacent protofilament. Our investigation of the designability of this arginine clasp motif suggested that it does not appear to have been evolutionarily sampled as a mechanism to mediate helix-helix interactions in native protein structures and cannot be considered as natively designable. Nevertheless, the RxxxR motif appeared robust as a design element within a structurally related family of cross α-helical assemblies and was preferred to other more designable interaction motifs, such as the common LxxxL motif, within an otherwise identical peptide sequence context. We demonstrated that within a designed series of coiled-coil peptides that the RxxxR motif could be employed to control nanotube structure through peptide length. The absence of the RxxxR motif drove the assembly down an alternative pathway, which suggested that the arginine clasp interaction was not robust outside of this relatively limited sequence context^12^.

From this perspective, we propose that the RxxxR clasp may be representive of a more general class of local interaction motifs that can potentially mediate quaternary interactions within a specific structural context in peptide and protein assemblies^18^. The influence of these localized interaction motifs may be magnified due to the presence of multiple copies in a structurally similar context along the contour length of the helical assembly, which enables selection for specific modes of self-association of protofilaments into higher order structures. Similar local interaction motifs, e.g., Phe-Phe diads^86–88^ and glutamine/asparagine ladders^83,89^, among others, have been identified that mediate higher-order interactions in cross-β assemblies. The relatively low information content of such short sequence motifs hinders a systematic sequence-based analysis of their role in higher order assembly processes. The challenge will be to effectively identify such motifs in order to exploit them for de novo design of helical assemblies. This process requires the application of high resolution structural analysis in order to identify the presence of such structurally determinative interactions. Cryo-EM is the primary method for structural characterization of helical filaments. More broadly applied structural investigations of naturally occurring and synthetic peptide and protein filaments represent the most promising approach to identify local structural interactions that contribute to specificity and stability within these assemblies.

## Methods

### Peptide synthesis and purification

Chemical reagents were purchased from Sigma-Aldrich Chemical Co. (St. Louis, MO) or Anaspec, Inc. (Fremont, CA) unless otherwise specified. Peptides 15-10-3, 22-17-3, 29-24-3, 36-31-3, 36-31-3_RR, 36-31-3_LL and 29-20-2 were obtained from SynPeptide Co., LTD. (Shanghai, China). All purchased chemical reagents and peptides ordered were used without further purification. The Form II and Form IIa peptides were prepared via microwave-assisted solid phase peptide synthesis on a CEM Liberty Blue Automated Microwave Peptide Synthesizer as the N-acetyl, C-amide capped derivatives. A PAL-PEG-PS resin from Applied Biosystems (Foster City, CA) was used for synthesis of both peptides. Standard Fmoc protection chemistry was utilized in conjunction with coupling cycles consisting of HBTU/OXIMA-mediated activation protocols and base-induced deprotection (20% piperidine in N, N*-*dimethylformamide with 0.1 M hydroxybenzotriazole) of the Fmoc group. After synthesis, the DMF/resin mixture was filtered and rinsed with acetone then air-dried. The peptides were cleaved from the resin by incubation at room temperature for 3 h in a cocktail consisting of 92.5% trifluoroacetic acid (TFA), 2.5% distilled water, 2.5% triisopropylsilane, and 2.5% 2,2’- (ethylenedioxy)-diethanethiol. Cleavage was followed by filtration and subsequent precipitation in diethyl ether. The precipitate was allowed to desiccate overnight. The crude peptides were resolubilized in 3 mL of a 50:50 mixture of acetonitrile and water (0.1% TFA additive) and purified using a Shimadzu LC-20AP reversed-phase high-pressure liquid chromatography (HPLC) equipped with a C18 column. The peptides were eluted with a linear gradient of water-acetonitrile with 0.1% TFA. Peptide mass was confirmed using ESI mass spectrometry. Purified HPLC fractions were lyophilized, sealed, and stored at −30 °C.

### Peptide assembly

Stock solutions of all peptides (3 mg/mL) were prepared by solubilizing purified, lyophilized peptide (0.75 mg) in 250 μL of acetate buffer (10 mM, pH 4.0). The solution was titrated with dilute sodium hydroxide solution to adjust the final pH value to 4.0. The solutions were allowed to assemble from 24 h to 2 weeks depending on the sequence. Peptides 36-31-6_RR and 36-31-6_LL were thermally annealed using the following thermal cycle protocol: (1) rapid heating to 90 °C for 30 min and (2) cooling to 25 °C at a rate of 0.2 °C/minute.

### Circular dichroism spectropolarimetry

CD measurements were performed on a Jasco J-1500 CD spectropolarimeter using 0.10 mm thick quartz plates (Hellma Analytics). Three CD spectra were collected and averaged for each peptide sample. Each spectrum was acquired at a scan rate of 100 nm/min from 190-260 nm with a bandwidth of 2 nm and a data pitch of 0.2 nm.

### Negative stain TEM analysis

TEM Grids were prepared using dilute solutions of peptide (3 mg/mL) in aqueous buffer (10 mM acetate, pH 4.0). Samples were prepared by depositing a peptide solution (4 μL) onto a 200-mesh carbon-coated copper grid from Electron Microscopy Services (Hatfield, PA). After 90 s of incubation on the grid, the excess liquid was wicked away, leaving a thin film of sample. An aliquot (4 μL) of negative stain solution (1% uranyl acetate) was deposited onto the thin film. After 1 min of staining, the remaining moisture was wicked away, and the grid was dried overnight in a desiccator. Electron micrographs were captured on a Hitachi HT-7700 transmission electron microscopy, equipped with a tungsten filament and AMT CCD camera, operating at an accelerating voltage of 80 kV.

### Cryo-EM image analysis

The peptide sample (ca. 2–4 μL) was applied to glow-discharged Quantifoil (1.2/1.3 or 2/2) or lacey carbon grids, and then plunge frozen using a Vitrobot Mark IV (FEI). Cryo-EM data sets on peptides 15-10-3, 29-24-3, 29-20-2 and 36-31-3_RR were collected on a 200 keV Talos Arctica with a K2 camera (Emory University) at 1.04 Å/pixel and a total dose of ca. 55 e/Å^2^. The cryo-EM images for the dataset of peptide 36-31-3 were collected on a 300 keV Titan Krios with a K2 camera (National Cryo-EM Facility at NCI) at 1.06 Å/pixel and a total dose of ca. 50 e/Å^2^. Cryo-EM datasets on peptides Form II and Form IIa were collected on a 300 keV Titan Krios with a K3 camera (University of Virginia) at 1.08 Å/pixel and a total dose of ca. 51 e/Å^2^. Cryo-EM movies were recorded in counting mode using EPU v2.4 (Thermo Fisher). The micrographs were first motion corrected and dose weighted by MotionCorr v2^90^, and then a CTF correction was applied by multiplying the images with the theoretical CTF using CTFFIND3. Filament images corresponding to ca. 20 electrons/Å^2^ were extracted using e2helixboxer (EMAN2)^91^. For each peptide filament, a list of possible helical symmetries was calculated from the averaged power spectrum of peptide particles. To determine the correct helical symmetry from the list, first if the full dataset has more than 30,000 particles, a subset containing 30,000 particles was generated. And then the initial volume was generated from those 30,000 particles with random assigned azimuthal angles. After that, possible helical symmetries were tested in Spider v22.10 using IHRSR^67,92^ by trial and error until recognizable amino acid side chain densities could be observed in the correct symmetry (see Supplementary Figs. 11 and 12 for representative examples of this process). Specifically, the IHSRS reconstructions were divided into 3 steps, where 4x binned, 2x binned, un-binned particles were used to accelerate the progress. Helical symmetries were tightly locked in the first two steps, and then slightly relaxed in the third step. All helical parameters, including rise, rotation and point-group symmetry were imposed at the end of each IHRSR cycle. After determining the correct symmetry, final reconstructions using the all particles were run in both RELION^93^ v3.0 and SPIDER^92^ v22.10. The resulting volumes from both approaches were sharpened with the same negative B-factor, and then selected by eye based on the quality of the side chain densities. The statistics are listed in Supplementary Tables 1 and 2.

### Model building

As the cryo-EM maps of all peptide samples reach to 4.5-Å resolution or higher, right-handed α-helices were used to determine the helical hand of the EM volumes. It was clear from the map that each peptide molecule forms a single α-helix. Therefore, an α-helix model was generated in UCSF Chimera^94^ v1.14 from the corresponding peptide sequence and then docked in the respective EM map. This single α-helix model was adjusted manually in Coot^95^ v0.8 to best fit into the map. Finally, this adjusted single α-helix model was used to generate a model filament using the determined helical symmetry, which was then refined against the full cryo-EM map using real space refinement in PHENIX^95^ v1.16. Final geometries of the atomic models were validated with the MolProbity^96^ implementation in PHENIX. The refinement statistics are shown in Supplementary Tables 1 and 2. Cryo-EM maps and atomic coordinates have been deposited with the Electron Microscopy Data Bank and Protein Data Bank with accession codes given in Supplementary Tables 1 and 2. Model versus map FSC calculations were employed to estimate the resolution of the reconstructions and are reported in Supplementary Fig. 13.

### Motif analysis

A search database was created by filtering the PDB, as of 04/19/2020, for the following: X-ray structures with resolution of 2.6 Å or better, containing at least 60% protein residues, with no more than 5000 residues and no more than 26 chains in the biological unit. This resulted in a total of 146,052 biological-unit entries. All-to-all sequence clustering of chains within these biological units was then performed at 50% sequence identity using the USEARCH tool v10^97^. Using this information, a minimally non-redundant subset of biological units was selected by eliminating a biological unit A if another one in the set contained a super set of its chains (i.e., had chains belonging to the same clusters as chains of A and additional chains). Resolution was used as the tie-breaker when two biounits had equivalent sets of chains. In the end, 23,915 biounits survived with a total of 176,442 chains and 43,449,344 residues. This set of structures was used to search for the Arg clasp structural motif (or the control motif) using the MASTER program^98,99^. All non-redundant matches (i.e., less than 50% sequence identity in the matching region—the matching fragments themselves + /- 15 residues on each side) with backbone RMSD below 1.0 Å were sought and used for analysis.

### Computational design

dTERMen^74,75^ was used to perform design. dTERMen is a general-purpose method for computational protein design that is based on the concept of structural degeneracy of proteins. Central to this approach is the concept of a TERM (short for tertiary motif)—a fragment of protein structure–consisting of one or more disjoint structural segments, that is reused across unrelated proteins. This reuse, which we described in several studies^100–102^, allows one to directly mine the PDB for sequence-structure relationships. Specifically, repeated TERM instances allow one to identify sequence features necessary for stabilizing the corresponding structural motif. Thus, dTERMen works by automatically breaking the desired target structure *T* down into its constituent TERMs, deducing sequence-structure relationships for each using rapid database searches (via the underlying method MASTER^98^) and combines these to build a model for what sequences would most likely stabilize target structure *T*.

A python-based implementation of dTERMen was employed in this study, which makes extensive use of the structure search engine MASTER to identify TERM matches and extract their sequence statistics. The structural database for underlying MASTER searches used in this study was prepared on 01/22/2019 following the procedure above. As the design template, a 15-chain portion of the 36-31-3 assembly was used (shown in color in Fig. 7c), with one chain (chain A) fully surrounded by all symmetry mates with which it could make any appreciable interactions. The symmetry-based design feature in dTERMen was used (–image flag), with chain A as the central unit and all others being images. The energy table resulting from running dTERMen described the sequence landscape compatible with folding to the 36-31-3 geometry, based on structural statistics of constituent motifs. Given this table, integer linear programing (ILP) was used to identify the best-scoring sequence under a solubility/fold-specificity constraint, requiring solvent-facing *g* positions (i.e., 8, 15, 22, and 29) to be polar (i.e., one of Asp, Glu, Gly, His, Lys, Asn, Gln, Arg, Ser, Thr, Tyr). The resulting sequence, TLEELLAEALILKAKAEILKAKAEVLKAKAEILKAL, lacked the Arg clasp motif, which was not surprising in light of our analysis of this motif’s natural designability. To test the importance of this motif for assembly formation, we generated another design by re-running the optimization with the additional constraint that preserved the Arg clasp motif (i.e., left identities of positions 6, 7, 10, and 36 at Arg, Ala, Arg, and Gln, respectively). This resulted in the sequence: TLEELRAEARILEAKAEILKAKAEVLKAKAEILKAQ, which was called 36-31-3_RR, while the original design was called 36-31-3_LL.

### Reporting summary

Further information on research design is available in the Nature Research Reporting Summary linked to this article.

## Supplementary information

Supplementary Information

Reporting Summary

## Data Availability

The reconstruction maps were deposited in the Electron Microscopy Data Bank with accession numbers of EMD-21812, EMD-21813, EMD-21814, EMD-21815, EMD-21816, EMD-21817, and EMD-21818. The corresponding filament models were deposited in the Protein Data Bank with accession numbers of 6WKX, 6WKY, 6WL0, 6WL1, 6WL7, 6WL8, and 6WL9. Additional data that support the findings of this study are available from the corresponding author upon request.

## Code availability

The codes for MASTER and dTERMen are freely available for noncommercial purposes from the Grigoryan Lab at Dartmouth College through the following links; https://grigoryanlab.org/master and https://grigoryanlab.org/dtermen, respectively.
