# Supplementary material for: Structural analysis of cross α-helical nanotubes provides insight into the designability of filamentous peptide nanomaterials
Source: Nat Commun. 2021 Jan 18;12:407. doi: 10.1038/s41467-020-20689-w (PMC7814010; doi:10.1038/s41467-020-20689-w)
Supplement: Supplementary file 1 — Supplementary Information [file 41467_2020_20689_MOESM1_ESM.pdf]

Supplementary Information

Structural analysis of cross α-helical nanotubes provides insight into the designability of filamentous peptide nanomaterials

Fengbin Wang, Ordy Gnewou, Charles Modlin, Leticia C. Beltran, Chunfu Xu, Zhangli Su, Puneet Juneja, Gevorg Grigoryan, Edward H. Egelman, Vincent P. Conticello*

* Correspondence to vcontic@emory.edu

Supplementary Figures

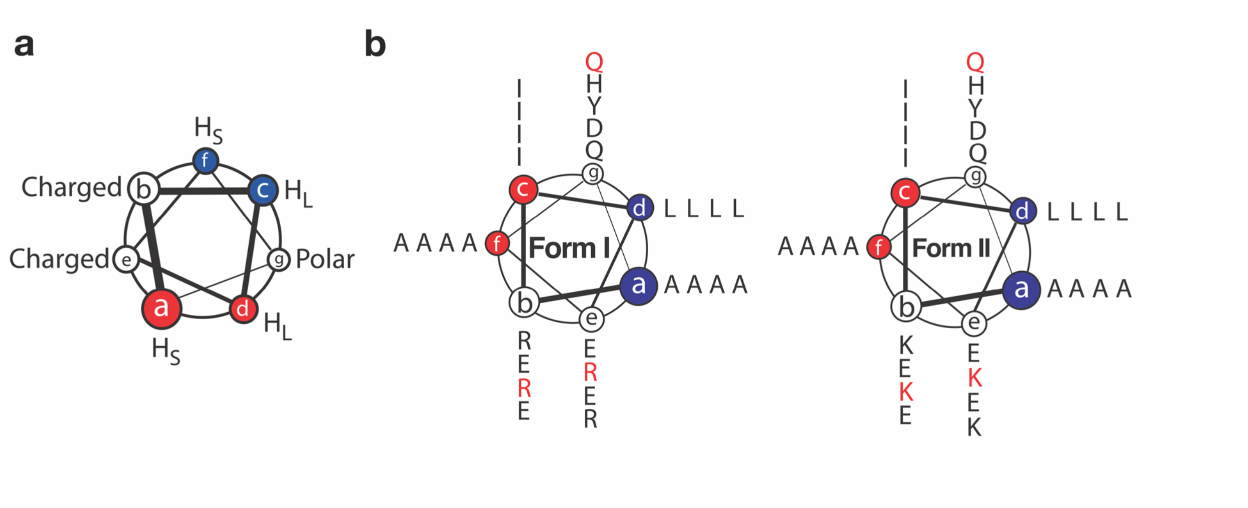

Supplementary Fig. 1 | Helical wheel diagrams representing peptide sequence designs in coiled-coil space. **a**, General design of Form peptide sequences based on the heptad repeat pattern of Type III coiled-coils. The two offset hydrophobic faces corresponding to the *a/d* and *c/f* residues are highlighted in red and blue, respectively. Selective heterotypic association between the *a/d* and *c/f* faces results in a two residue displacement between interacting peptides along the helix-helix interface. (H_L, large hydrophobic residues; H_S, small hydrophobic residues) **b**, Heptad sequences of the Form I and Form II parent peptides threaded onto a coiled-coil helical wheel diagram. The residues involved in the arginine clasp interaction (R13, R17, Q29) are highlighted in red for the Form I sequence, as well as the corresponding positions in the Form II sequence.

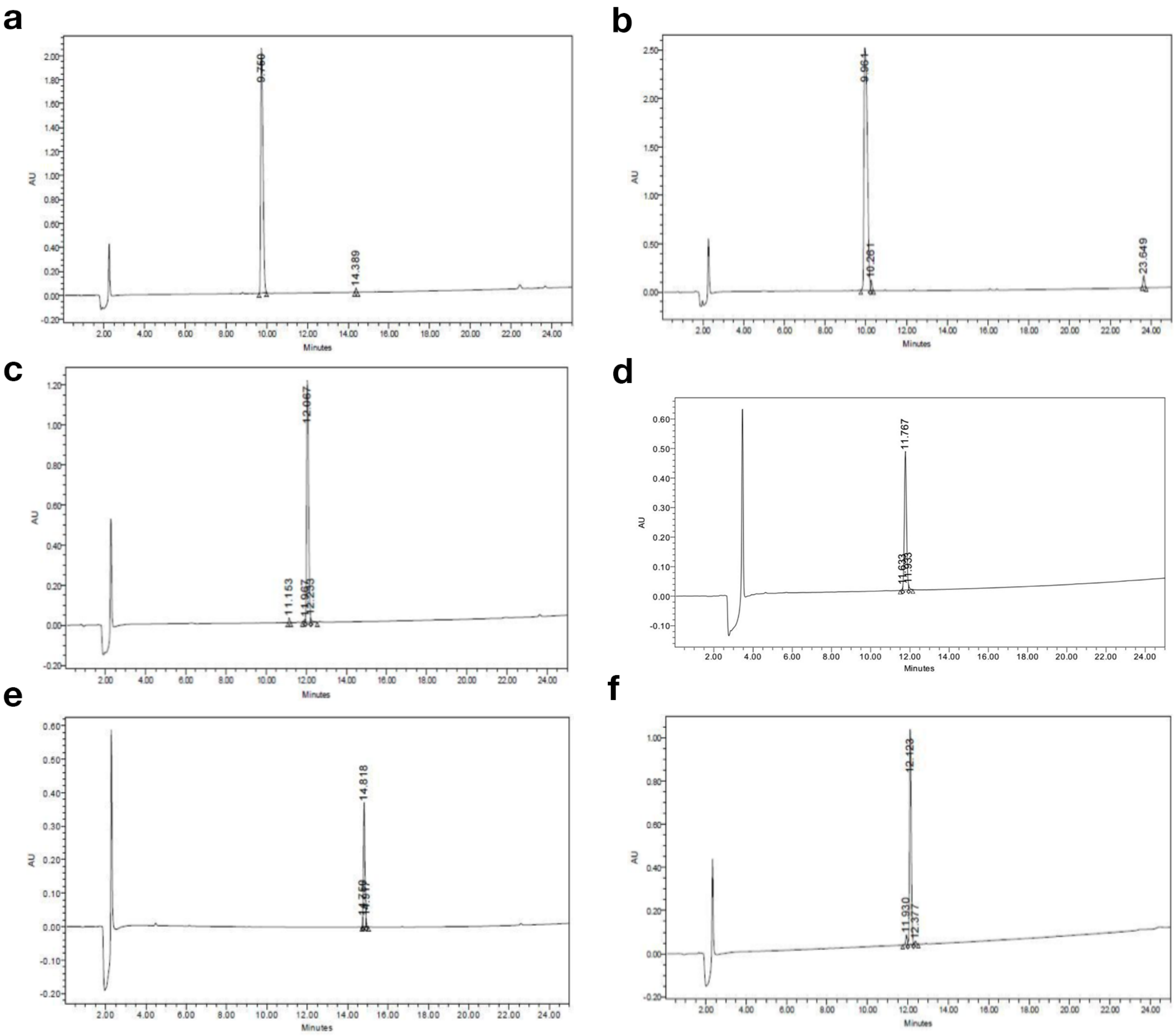

Supplementary Fig. 2 | Analytical HPLC traces of Form I-like peptides (**a**, 15-10-3; **b**, 22-17-3; **c**, 29-24-3; **d**, 36-31-3; **e**, 36-31-3_LL; **f**, 36-31-3_RR).

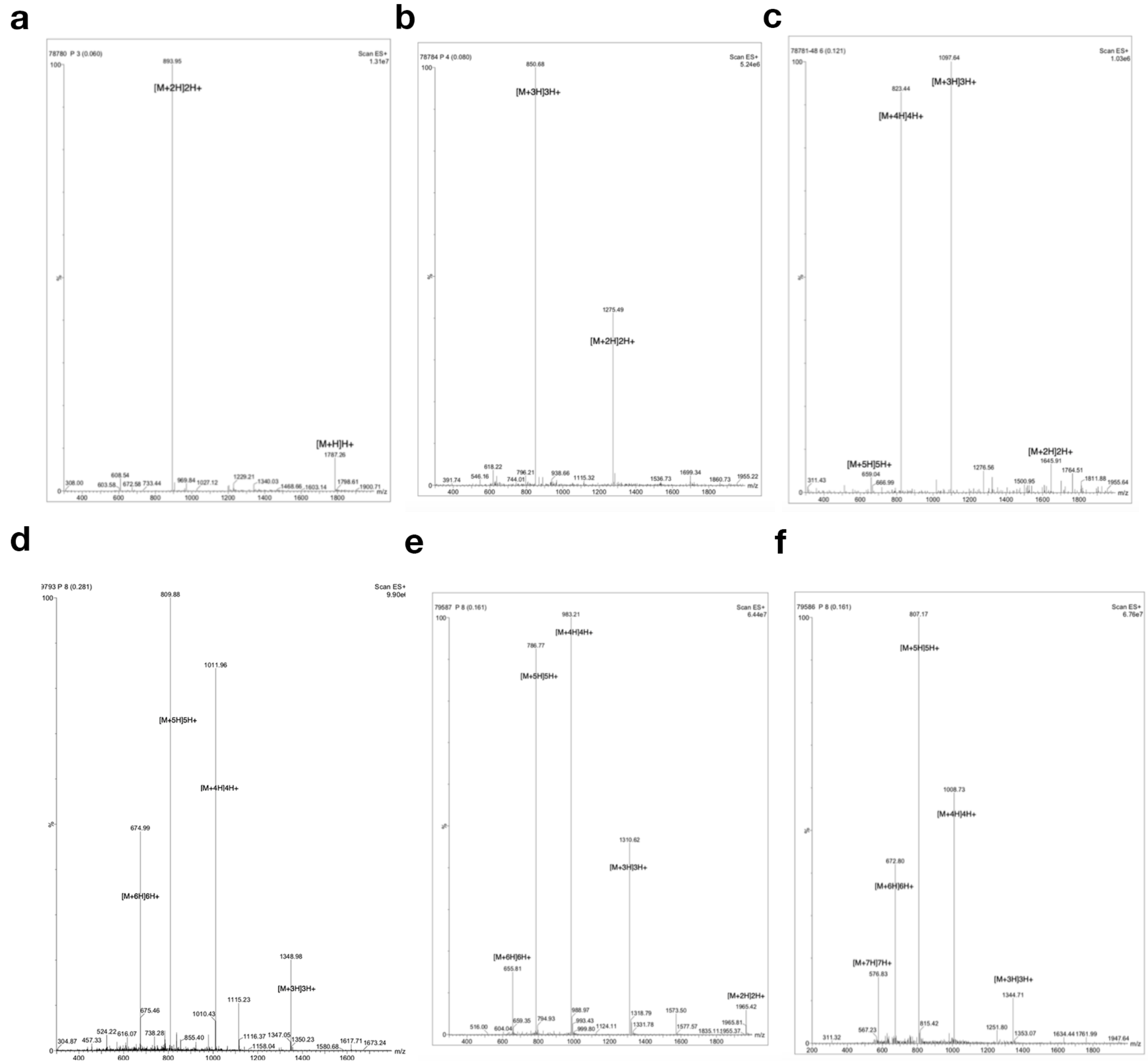

Supplementary Fig. 3 | Electrospray mass spectra of Form I-like peptides (**a**, 15-10-3; **b**, 22-17-3; **c**, 29-24-3; **d**, 36-31-3; **e**, 36-31-3_LL; **f**, 36-31-3_RR).

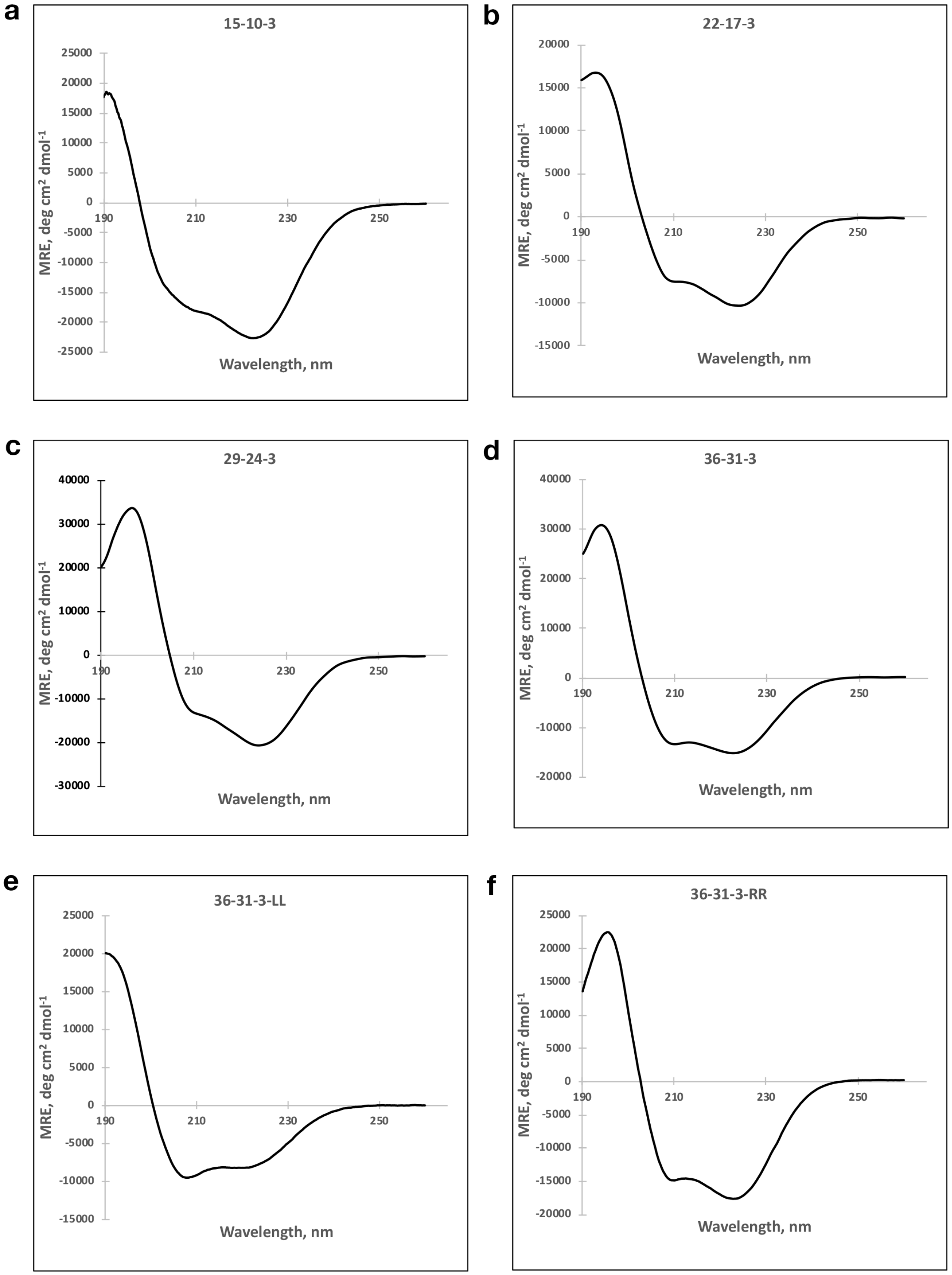

Supplementary Fig. 4 | Circular dichroism spectra of Form I-like peptides (**a**, 15-10-3; **b**, 22-17-3; **c**, 29-24-3; **d**, 36-31-3; **e**, 36-31-3_LL; **f**, 36-31-3_RR).

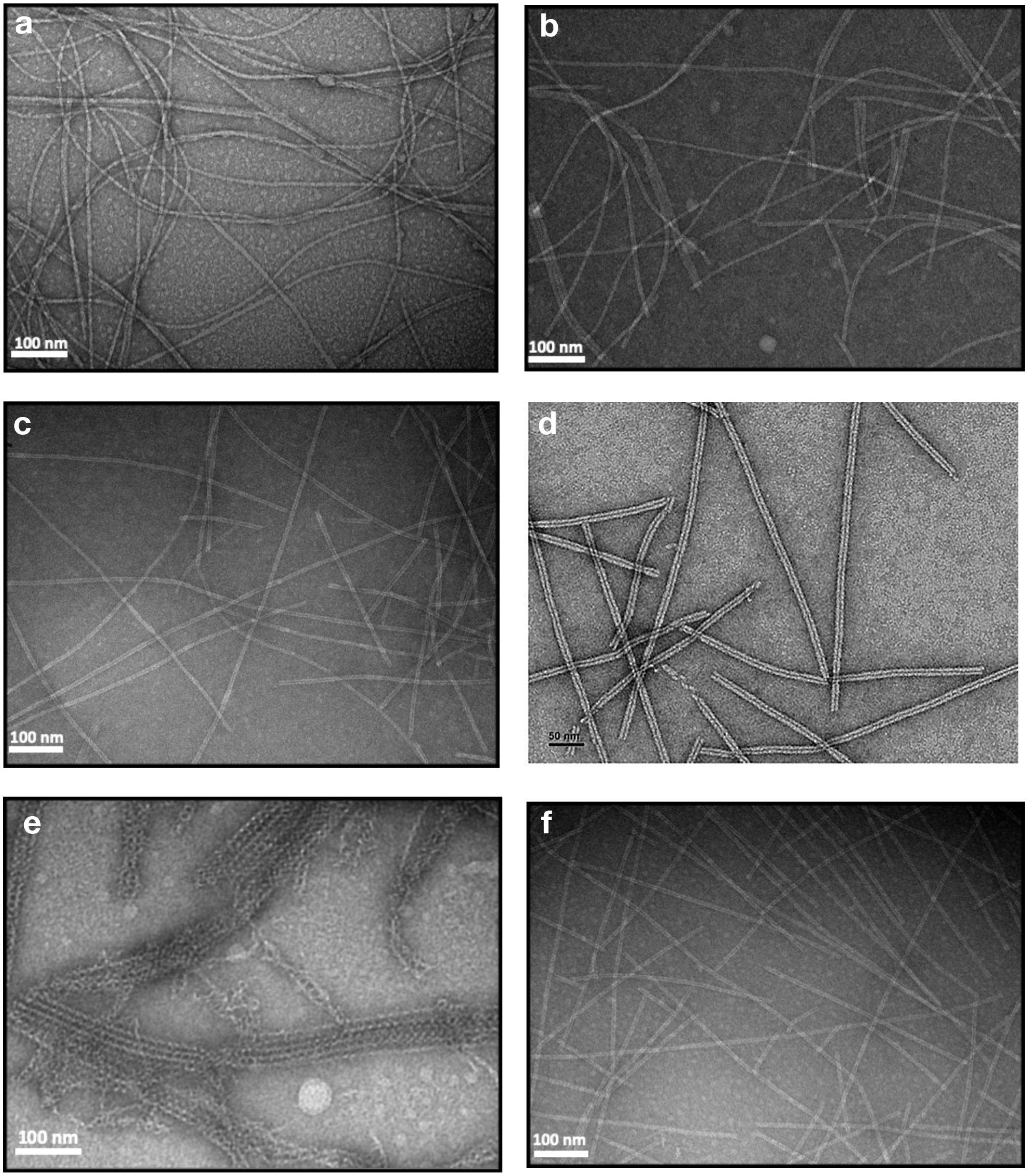

Supplementary Fig. 5 | Representative negative-stain TEM images of Form I-like peptides (**a**, 15-10-3; **b**, 22-17-3; **c**, 29-24-3; **d**, 36-31-3; **e**, 36-31-3 LL; **f**, 36-31-3 RR). Total number of collected TEM images for each sample: 30 (15-10-3); 30 (22-17-3); 30 (29-24-3); 14 (36-31-3); 10 (36-31-3_LL); 15 (36-31-3_RR).

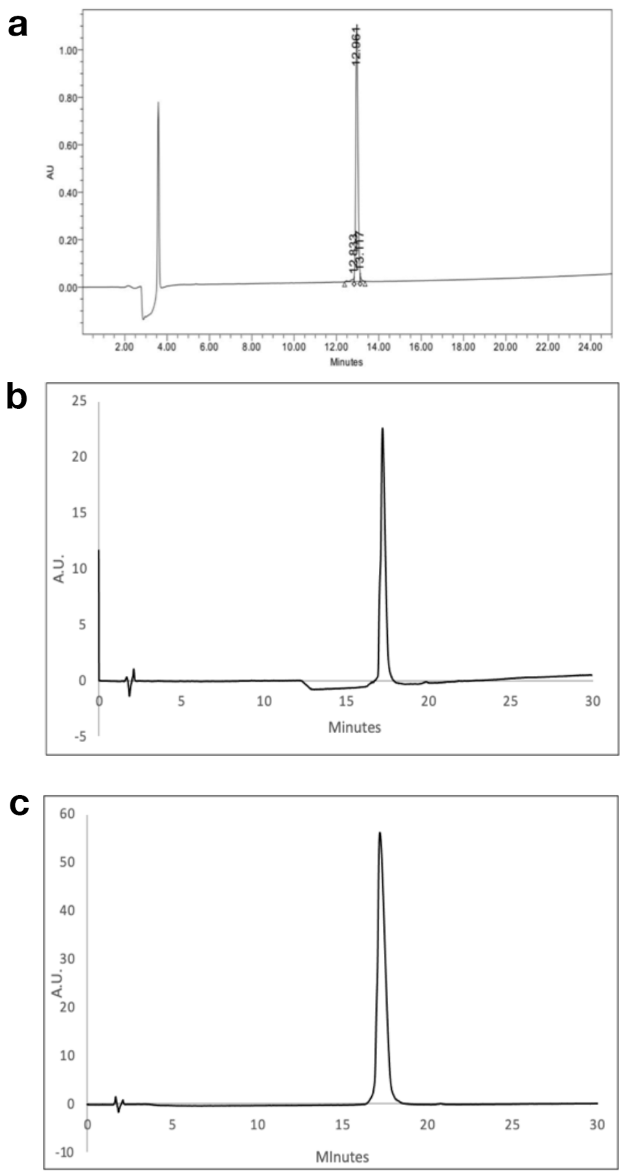

Supplementary Fig. 6 | Analytical HPLC traces of Form II-like peptides (**a**, 29-20-2; **b**, Form II; **c**, Form IIa).

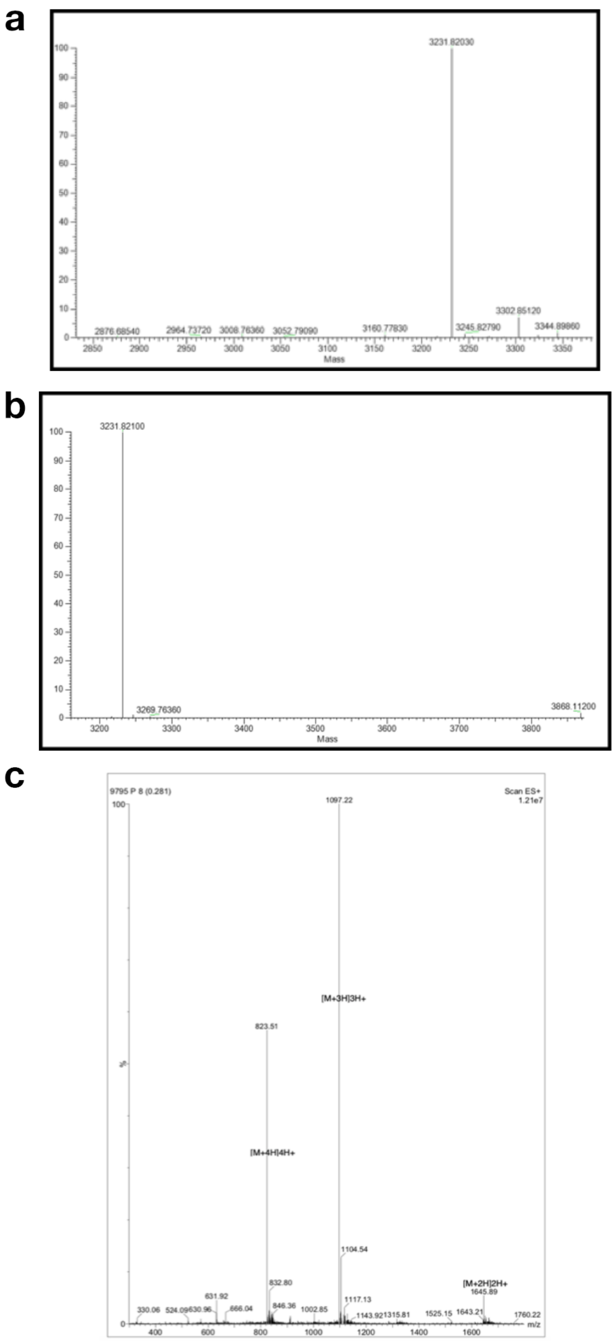

Supplementary Fig. 7 | Electrospray mass spectra of Form II-like peptides (**a**, Form II; **b**, Form IIa; **c**, 29-20-2).

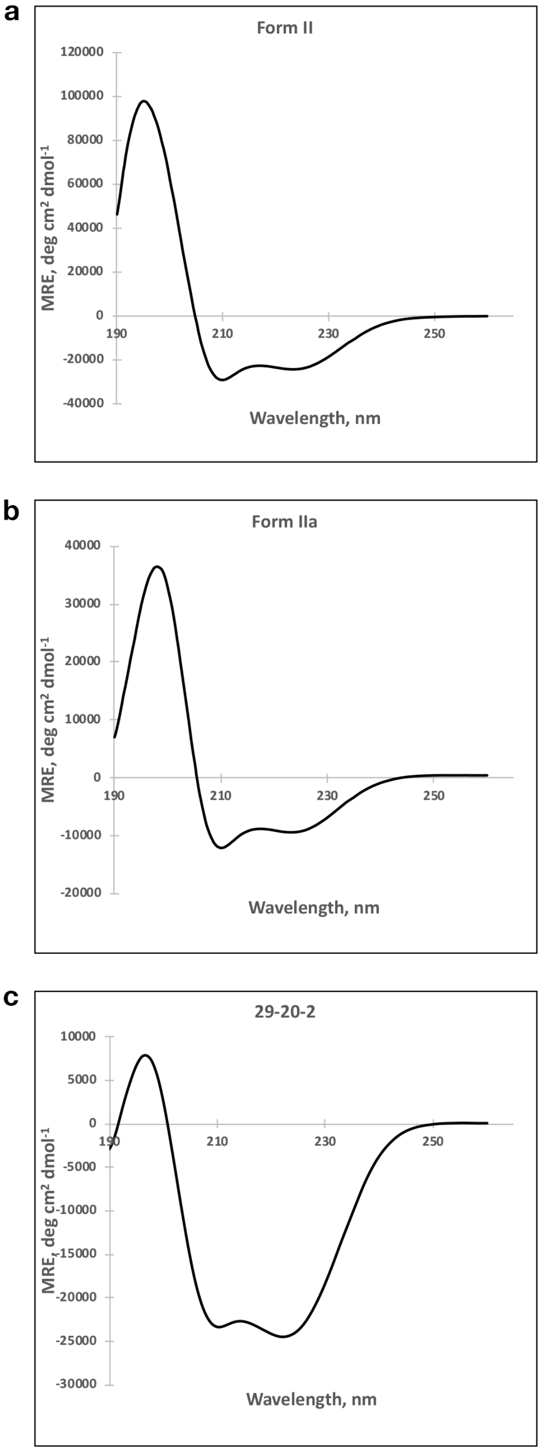

Supplementary Fig. 8 | Circular dichroism spectra of Form II-like peptides (**a**, Form II; **b**, Form IIa; **c**, 29-20-2).

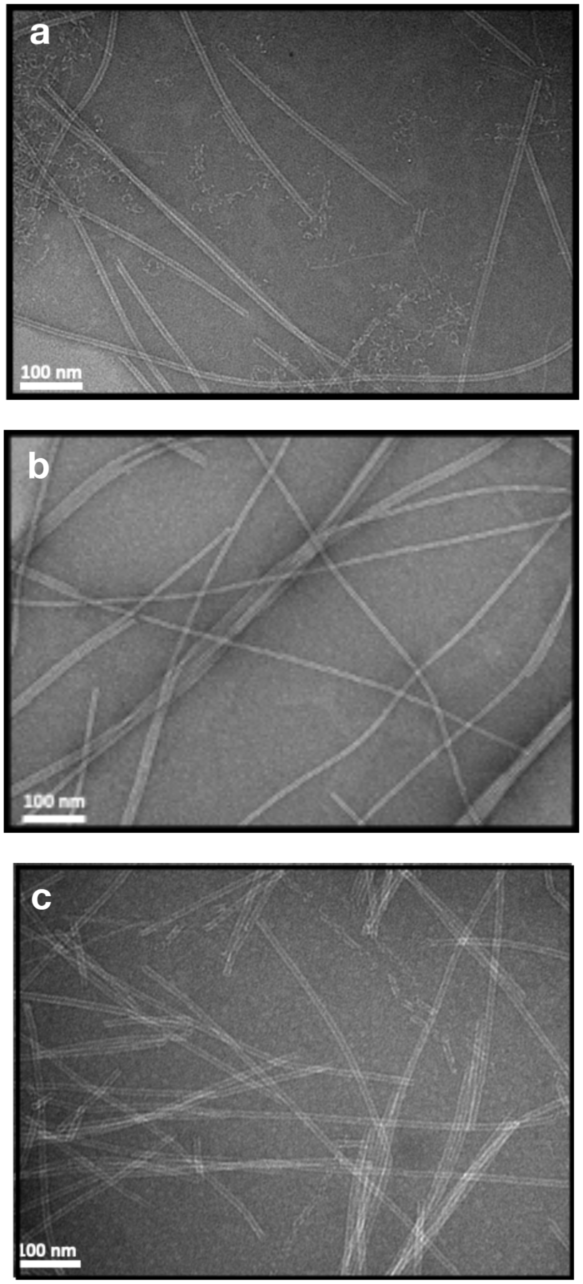

Supplementary Fig. 9 | Representative negative stain TEM images of Form II-like peptides (**a**, Form II; **b**, Form IIa; **c**, 29-20-2). Total number of collected TEM images for each sample: 12 (Form II); 12 (Form IIa); 20 (29-20-2).

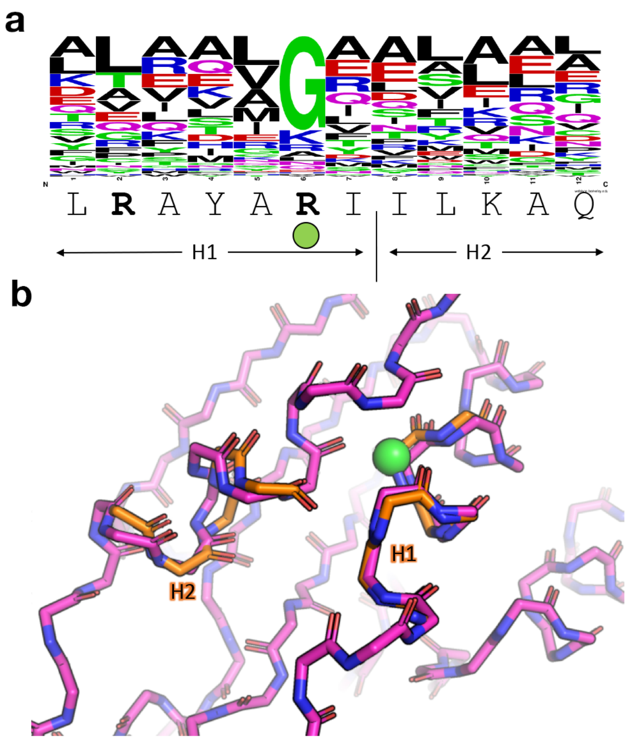

Supplementary Fig. 10 | Comparison between the arginine clasp motif and closest non-redundant matches. **a**, Sequence logo of the closest non-redundant sequence matches to the Arg clasp fragments. The green dot indicates the position of the conserved glycine residue in helix 1 (H1), which occurs in place of the second arginine in the RxxxR motif. Mutagenesis of the arginine at this position in Form I-like structures abrogates the arginine clasp interaction and results in conversion to Form II-like structures. **b**, Structural overlay of the interacting segments of the arginine clasp motif (H1 and H2, orange) in the 36-31-3 structure with the closest match (pink). The latter occurs in the context of an internal helix-helix interaction of two closely-approaching helices, which necessitates the presence of a glycine residue (green dot).

a

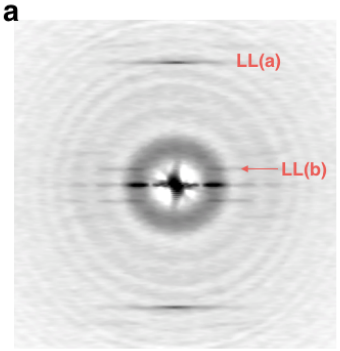

b

Tested symmetries	Bessel order		Calculated helical symmetry			IHRSR volumes	Rod-like features?	See side chains?	Correct symmetry?
	LL(a)	LL(b)	Rise (Å)	Twist (°)	Point group				
#1	0	+3	9.2	15.3	C3	panel (c)	No	No	No
#2	0	+4	9.2	11.5	C4	panel (d)	No	No	No
#3	0	+5	9.2	9.3	C5	panel (e)	Yes	Yes	Yes
#4	0	+6	9.2	7.7	C6	panel (f)	No	No	No

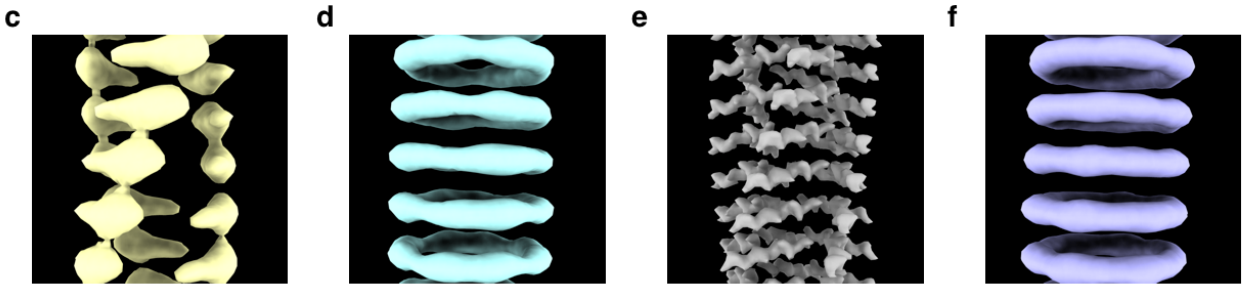

Supplementary Fig. 11 | Helical symmetry determination of Form I-like peptide, 15-10-3. **a,** Averaged power spectrum of the segments used in the initial reconstruction. The layer lines (LL) that were used to calculate the helical symmetry are labelled as LL(a) and LL(b). **b,** List of tested symmetries. The Bessel order assigned for each symmetry, detailed helical parameters, and additional criteria to judge the results. **c-f,** The resulting IHRSR volumes of tested symmetries listed in panel (**b**)

a

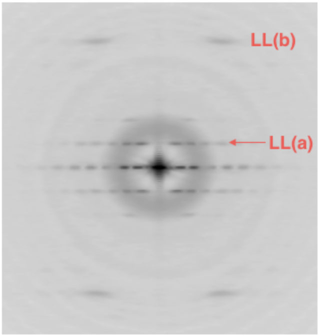

b

Tested symmetries	Bessel order		Calculated helical symmetry			IHRSR volumes	Rod-like features?	See side chains?	Correct symmetry?
	LL(a)	LL(b)	Rise (Å)	Twist (°)	Point group				
#1	+3	-9	6.28	14.5	C3	panel (c)	Yes	No	No
#2	+3	-10	2.02	-115.35	C1	panel (d)	No	No	No
#3	+3	-11	1.95	124.48	C1	panel (e)	Yes	No	No
#4	+3	-12	5.63	12.95	C3	panel (f)	Yes	Yes	Yes

c d e f

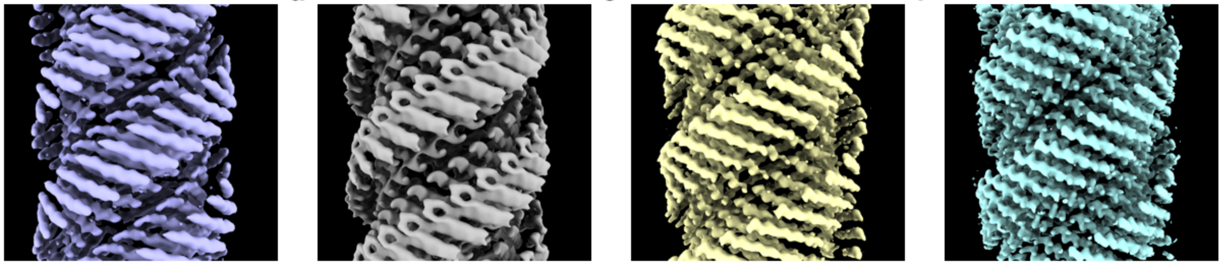

Supplementary Fig. 12 | Helical symmetry determination of the Form II-like peptide, 29-20-2. **a,** Averaged power spectrum of the segments used in the initial reconstruction. The layer lines (LL) that were used to calculate the helical symmetry are labelled as LL(a) and LL(b). **b,** List of tested symmetries. The Bessel order assigned for each symmetry, detailed helical parameters, and criteria to judge the results. **c-f,** The resulting IHRSR volumes of tested symmetries listed in panel (**b**)

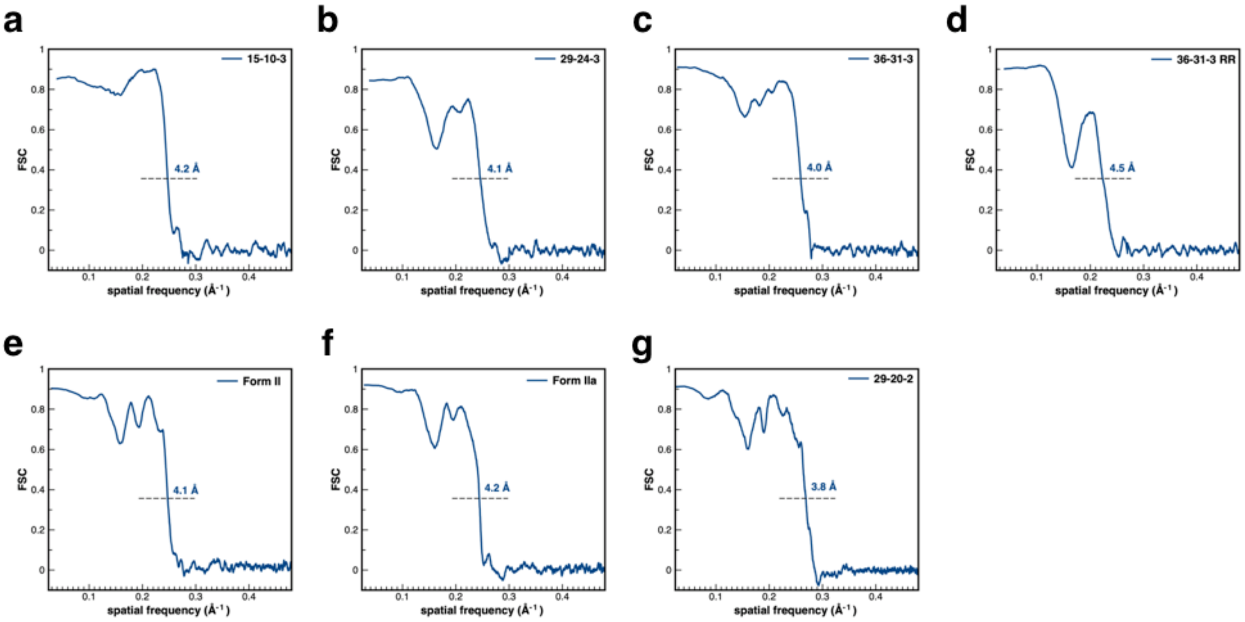

Supplementary Fig. 13 | Fourier Shell Correlation (FSC) calculations. The model:map FSC calculation using a 0.38 criterion, which is sqrt (0.143) to estimate the resolution. (**a**, 15-10-3; **b**, 29-24-3; **c**, 36-31-3; **d**, 36-31-3 RR; **e**, Form II; **f**, Form IIa; **g**, 29-20-2).

Supplementary Tables

Supplementary Table 1. Cryo-EM and Refinement Statistics of Form I Related Nanotubes

Parameter	15-10-3	29-17-3 (Form I)*	29-24-3	36-31-3	36-31-3 RR
**Data collection and processing**					
Voltage (kV)	200	300	200	300	200
Electron exposure ($e^{-}Å^{-2}$)	55	50	55	50	55
Pixel size (Å)	1.04	1.02	1.04	1.06	1.04
Final particle images (n)	11,334	62,122	12,869	66,079	39,805
**Helical symmetry**					
Point group	C5	C1	C4	C1	C1
Helical rise (Å)	9.21	2.20	7.96	2.50	2.51
Helical twist (°)	9.3	-87.1	10.9	124.0	124.5
**Map resolution (Å)**					
Model:map FSC (0.38)	4.2	3.6	4.1	4.0	4.5
d_{99}	4.3	3.7	4.2	4.0	4.6
**Refinement and Model validation**					
Map-sharpening B-factor ($Å^2$)	-100	-100	-120	-200	-120
Bond lengths rmsd (Å)	0.006	0.093	0.006	0.007	0.006
Bond angles rmsd (°)	0.557	1.875	0.726	0.797	0.810
Clashscore	2.5	9.2	7.2	2.0	6.3
Poor rotamers (%)	0	0	0	0	0
Ramachandran Favored (%)	100	96.5	99.4	100	100
Ramachandran Outlier (%)	0	0	0.6	0	0
MolProbity score	1.04	1.72	1.40	0.97	1.35
**Deposition ID**					
PDB (model)	6WKX	3J89	6WKY	6WL1	6WL0
EMDB (map)	EMD-21812	EMD-6123	EMD-21813	EMD-21815	EMD-21814

*Filament structure reported on 2015.

Supplementary Table 2. Cryo-EM and Refinement Statistics of Form II Related Nanotubes

Parameter	Form II	Form IIa	29-20-2
**Data collection and processing**			
Voltage (kV)	300	300	200
Electron exposure ($e^{-}Å^{-2}$)	51	51	55
Pixel size (Å)	1.08	1.08	1.04
Final particle images (n)	408,751	288,990	67,941
**Helical symmetry**			
Point group	C1	C1	C3
Helical rise (Å)	1.93	1.92	5.63
Helical twist (°)	124.36	124.37	12.90
**Map resolution (Å)**			
Model:map FSC (0.38)	4.1	4.2	3.8
d_{99}	4.4	4.6	4.1
**Refinement and Model validation**			
Map-sharpening B-factor ($Å^2$)	-189	-176	-100
Bond lengths rmsd (Å)	0.006	0.006	0.006
Bond angles rmsd (°)	0.805	0.530	0.559
Clashscore	2.1	14.7	8.5
Poor rotamers (%)	0	0	0
Ramachandran Favored (%)	100	98.2	98.8
Ramachandran Outlier (%)	0	0	0
MolProbity score	0.98	1.67	1.46
**Deposition ID**			
PDB (model)	6WL8	6WL9	6WL7
EMDB (map)	EMD-21817	EMD-21818	EMD-21816
